# A Primary Mesenteric Hydatid Cyst in an Adult Female Mimicking an Ovarian Cyst: The First Reported Case in Lebanon With Review of the Literature

**DOI:** 10.7759/cureus.89153

**Published:** 2025-07-31

**Authors:** Farah Assi, Douaa Abou Hamdan, Youmna Mourad, Ghenwa K El Dakdoukii, Leila Akil, Nada Chamseddine

**Affiliations:** 1 Infectious Diseases/Internal Medicine, Lebanese University Faculty of Medicine, St Georges Hospital, Beirut, LBN; 2 Infectious Diseases, Lebanese University Faculty of Medicine, Beirut, LBN; 3 Infectious Diseases, Lebanese University, Beirut, LBN; 4 Infectious Diseases, Bekaa Hospital, Salloum Medical Center, Bekaa, LBN; 5 Laboratory Medicine, Al Hadi Laboratory and Medical Center, Beirut, LBN; 6 Infectious Diseases, Beirut Arab University, Beirut, LBN; 7 Infectious Diseases/Internal Medicine, Hammoud Hospital University Medical Center, Sidon, LBN; 8 Pathology Medicine, Bahman University Hospital, Beirut, LBN; 9 Infectious Diseases/Internal Medicine, Zahraa University Medical Center, Beirut, LBN; 10 Infectious Diseases, Zahraa University Medical Center, Lebanese University, Beirut, LBN

**Keywords:** abdominal cystic masses, echinococcosis, hydatid cyst, ovarian cyst, primary mesenteric hydatid cyst

## Abstract

Hydatid cyst is a parasitic zoonosis caused by echinococcosis. Liver and lung are the two primarily affected organs. Primary mesenteric hydatid cyst is a rare event, and few cases have been reported globally. In this case report, we present the first documented case, to our knowledge, in Lebanon. The patient was a 49-year-old female who presented for acute worsening of a long-standing abdominal pain. The primary differential diagnosis was an ovarian cyst, for which urgent surgical excision was performed. Histopathological analysis showed a primary mesenteric hydatid cyst, thus providing the final diagnosis. This case highlights the diagnostic challenges that rare and atypical localizations of hydatid cysts present, and the importance of including echinococcosis in the differential diagnosis of abdominal cystic masses.

## Introduction

*Echinococcus* spp. is a pathogenic tapeworm, belonging to the family of Taeniidae, that leads to the development of a zoonotic disease termed hydatid cyst [1,2]. Humans, who serve as inadvertent intermediate hosts, become infected through the ingestion of foods contaminated with excrements of affected dogs, foxes, and wolves [3]. The cysts can expand to multiple different visceral organs, resulting in a wide range of localizations and clinical presentations [1]. Echinococcal disease manifests in two distinct forms: unilocular hydatid disease, caused by infection with *Echinococcus granulosus*, and alveolar hydatid cyst disease caused by infection with* Echinococcus multilocularis* [4]. Two other less commonly encountered species, *Echinococcus vogeli*, and *Echinococcus oligarthus*, are known to cause polycystic neotropical hydatid disease [4]. Cystic echinococcosis, caused by *E. granulosus*, is the most reported form, accounting for 95% of the estimated two to three million cases worldwide annually [5]. Besides humans, sheep and cattle are among the intermediate hosts, and since the transmission of *E. granulosus* spreads through domestic dogs in farming areas, echinococcosis is prevalent worldwide [1,3,4]. The liver is the most affected site (55%-70%), followed by the lungs (18%-35%) [6,7]. The occurrence of primary intrabdominal hydatid disease, without liver or lung affection, is infrequent, and among abdominal hydatidosis, primary mesenteric involvement that remains asymptomatic for years is rare [7,8], with a reported prevalence of 1:100,000 in adults [9].

The aim of this report is the describe the first reported case of primary mesenteric hydatid disease in Lebanon.

## Case presentation

We report the case of a 49-year-old female patient, not known to have any food or drug allergy, smoker, with a medical and surgical history positive for iron deficiency anemia, sinus tachycardia on bisoprolol, chronic migraine on pregabalin, knee cartilage repair, and C-section, who presented to the emergency room complaining of severe right lower quadrant pain radiating to the left lower quadrant and flank. The pain was associated with fever and nausea and increased in severity four days prior to presentation. She reported no urinary symptoms. Acute appendicitis was initially suspected. Upon further questioning, the patient mentioned a history of intermittent abdominal pain, present over the past four years, that was treated by multiple courses of antibiotics and painkillers without full improvement. This pain had been increasing in severity over the last two weeks until presentation. The patient was a housewife, living in a rural area, with no history of contact with dogs or cattle, and without any travel history. Upon presentation, the patient was afebrile with vital signs within normal limits. Physical exam showed a soft abdomen with right lower quadrant tenderness. There was no rebound tenderness, and the remaining physical exam was unremarkable. She was neurologically intact and had no skin rashes.

Labs at presentation demonstrated a normal leukocyte count, mild anemia, and mild elevation of C-reactive protein (CRP). Urine analysis was non-significant except for an alkaline pH. A summary of the initial laboratory tests are shown in Table 1.

**Table 1:** Laboratory data at admission WBC, white blood cells; Hb, hemoglobin; Plt, platelets; Cr, creatinine; C-RP, C-reactive protein; B-HCG: chorionic gonadotropin; LDH, lactate dehydrogenase; RBC, red blood cells; µl, microliter; hpf, high power field.

Parameter		Value	Reference range
WBC		9.07 x10³/µl	4.0-11.0/µl
Neutrophils		57.5%	40-65%
Hb		11 g/dl	12-16 g/dl (female)
Plt		240,000/µl	150-400/µl
Cr		0.69 mg/dL	0.4-1.1 mg/dL
C-RP		21.1 mg/L	0-5 mg/L
B-HCG		<1.2 mIU/mL	Negative 0-5 mIU/mL
LDH		136 IU/L	140-271 IU/L
Urine analysis: pH		7.5	5-6
Urine analysis: WBC		2-4/hpf	1-2/hpf
Urine analysis: RBC		0-2/hpf	0-1/hpf

**Figure 1:**
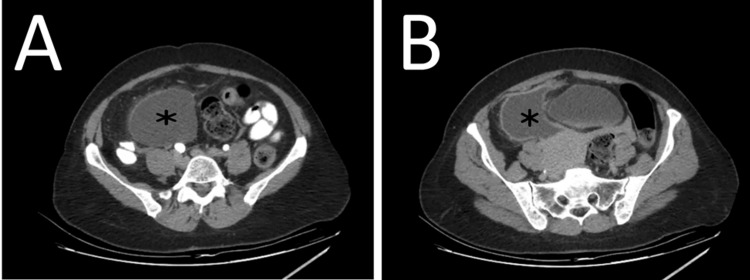
(A,B): A contrast-enhanced computed tomography (CECT) scan showing a well-defined 10-cm right infraumbilical hypodense mass showing a cystic density with peripheral enhancement (asterisks).

A contrast-enhanced computed tomography (CECT) scan of the abdomen and the pelvis was performed, which revealed a well-defined 10-cm right infraumbilical hypodense mass showing a cystic density with peripheral enhancement, surrounded by fat stranding. No appendicitis was seen on imaging. The remaining abdominal organs were without any pathology. The preliminary diagnosis was a complicated right ovarian cyst (Figure 1).

**Figure 2:**
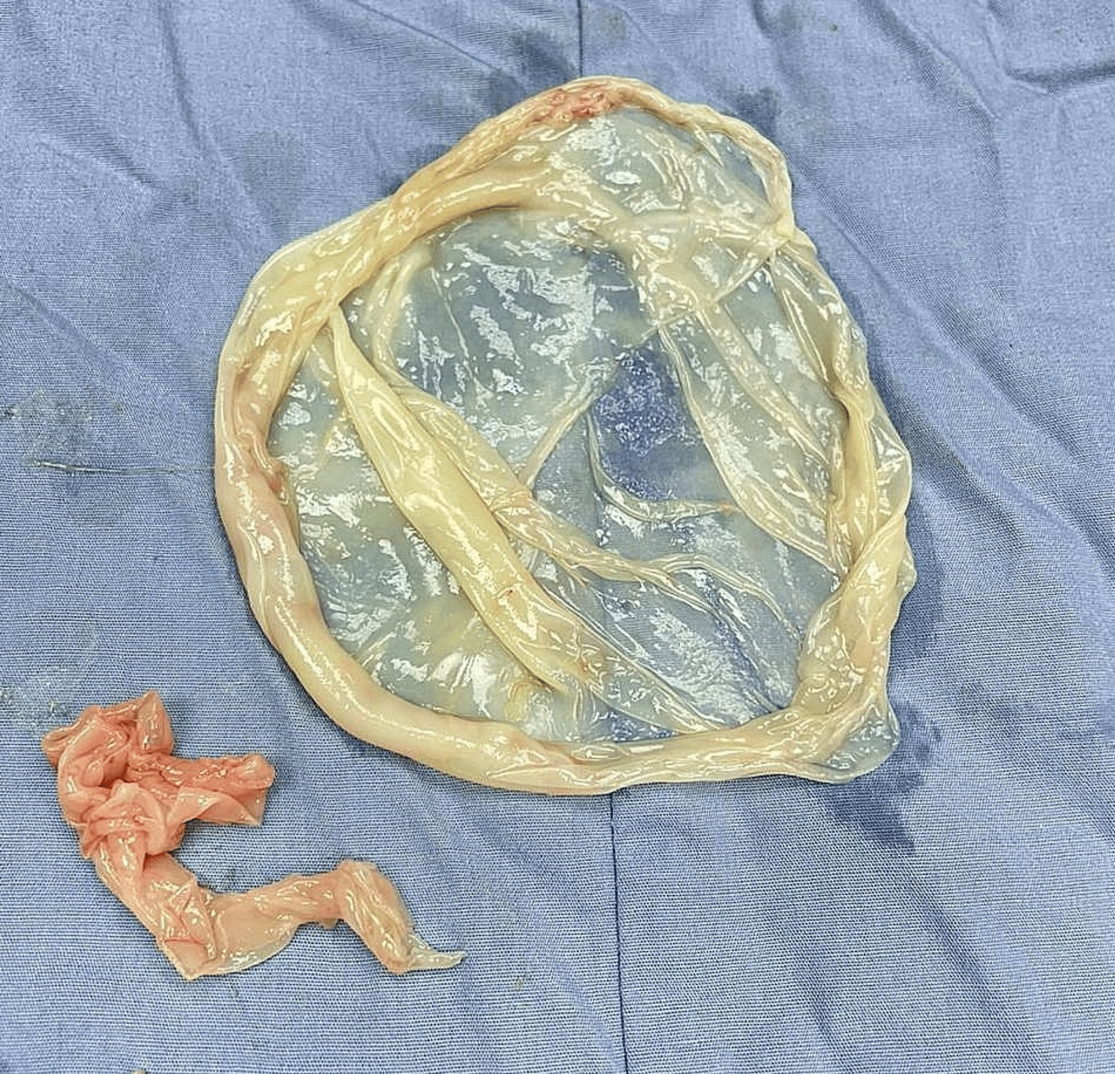
The wall of the mesenteric hydatid cyst removed in the operative room.

The patient was transferred to the operating room on an emergency basis for ovarian cystectomy by the obstetrical team. The operation was smooth without complication. Under general anesthesia and in the supine position, scrubbing and draping were performed and a Pfannenstiel incision was made. The abdomen was opened, layer by layer, until reaching the peritoneal cavity. Multiple intra-abdominal adhesions were found. The cyst was identified and found to be not originating from the ovary. An intermesenteric cyst was suspected. The cyst was dissected and removed (Figure 2) and then sent to the pathology lab. Hemostasis was done with closure of the wound layer by layer, and the patient was transferred to the recovery room.

**Figure 3:**
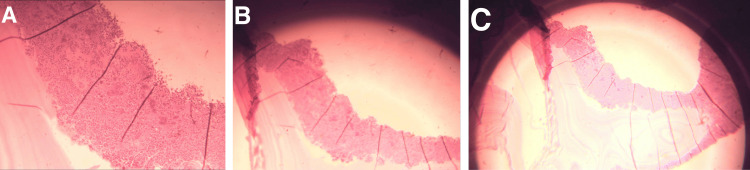
Histopathologic examination of the mesenteric hydatid cyst (A, B, and C).

On the first post-operative day, the patient was hemodynamically stable. She was started on prophylactic anticoagulation, cefazolin intravenously, proton pump inhibitor (PPI), and paracetamol for pain management. She was discharged 48 hours later and scheduled for follow-up. Her course in the hospital was smooth, and no post-operative complications were experienced. Five days later, the biopsy revealed a cystic wall made of fibrolamellar and hyalinized tissue filled with cells, consistent with a hydatid cyst. No ovarian stroma was seen and there was no evidence of malignancy (Figure 3). The patient was then started on oral albendazole 800 mg per day.

In the follow-up visit one month later, the patient was in a good clinical condition with no complaints. Albendazole was well tolerated with liver function tests within normal limits. Indirect hemagglutination (IHA) for hydatid serology level was low positive after three months of therapy (1.26; negative 0-0.9).

## Discussion

Two hosts are required in the life cycle of *E. granulosus* [7,9]. The adult worm lives in the proximal small bowel of the definitive host, usually a dog, where it excretes its eggs in the feces of the definitive host, contaminating the surrounding environment [2,10]. Resistant to desiccation, the eggs remain viable in the environment for weeks where humans and sheep, the recognized intermediate hosts, acquire echinococcosis through ingestion of foods contaminated with these shed eggs [2,3,10]. Once ingested by the intermediate host, the eggs reach the duodenum and release oncospheres that penetrate the intestinal mucosa, enter the portal circulation, and develop over time to form mature larval cysts [5,10,11]. The liver and lungs act as first and second filters, consecutively, explaining the frequent localization of cysts, with only 15% of the cases bypassing the liver and developing in other sites [5,8]. Following the liver and lungs, the oncospheres may migrate to the kidneys (3%), bones (1%-4%) and the brain (1%-2%) [6,7]. Other rarely affected organs are the heart, spleen, pancreas, omentum, ovaries, parametrium, pelvis, thyroid, orbit or retroperitoneum, and muscles [6,7]. Mesenteric hydatid disease, whether primary or secondary, constitutes one of the most rarely reported sites in the literature [9,10]. A solitary hydatid cyst located in the mesentery is only considered primary when no other cyst in other locations is discovered [4]. In this case, the oncosphere migrates directly through the hematogenous route or the lymphatics to reach the mesentery [4]. Clinically, symptoms of hydatid cysts in the mesentery are nonspecific and scarce and usually tolerated for prolonged periods of time [3]. The presentation depends on the size, the location, and the mass effects exerted by the cyst or cysts on adjacent organs [8]. Pain represents the most reported complaint and may be acute in onset or chronic, dull, or aching [3]. The presence of fever represents secondary cyst infection [3].

The diagnosis of hydatid cyst disease requires the presence of a low threshold of suspicion with a combination of available (both laboratory and radiological) tools [3,9,12]. The laboratory tools consist of serological tests, antigen detection tests, and polymerase chain reaction (PCR), such as complement fixation, IHA, latex agglutination, enzyme-linked immunosorbent assay (ELISA), Dot-ELISA, and many others [12]. Both ELISA and Dot-ELISA appear to provide better accuracy [12]. In a study by Maleki et al. Dot-ELISA, at a 1:800 dilution, showed 98.3% sensitivity and 100% specificity, making it a reliable and cost-effective diagnostic method. Purified antigen B had 96.7% specificity and 88.3% sensitivity, whereas crude hydatid fluid antigen had 93.3% sensitivity but only 76.7% specificity due to cross-reactions. ELISA using antigen B showed 89% sensitivity and 85.7% specificity, while IgG-based tests reached 97% sensitivity and 95.7% specificity [12]. The sensitivity and specificity of ELISA in diagnosing hydatid disease appear to be highest when the cyst is located in the liver; however, this sensitivity appears to decrease when the cyst is located in the lung (55%-56%), even more so when the cyst is located in other sites (25%-26%) [13]. Therefore, a negative ELISA test in extrahepatic sites does not exclude the diagnosis [13]. The complement fixation test is positive in approximately 65%, and the indirect hemagglutination test and ELISA have approximately 85% sensitivity [9].

Establishing a preoperative diagnosis of mesenteric hydatid disease is challenging, considering its rarity, lack of specific symptoms, its variable appearance on imaging modalities depending on its stage, and its resemblance to other cystic lesions in the abdominal cavity [14]. Ultrasound, CT scan, and magnetic resonance imaging (MRI) can detect the cyst preoperatively [7].

Two ultrasound-based staging systems are present to characterize and stage hydatid cysts: the World Health Organization (WHO) classification and Gharbi classification (Table 2) [15].

**Table 2:** Description of the WHO and Gharbi classification systems for staging of hydatid cysts. Source: Brunetti et al., 2010 [15]. CL: cystic lesion; CE: cystic echinococcosis.

Gharbi Classification	WHO Classification	Description	Stage
-	Type CL	Cystic lesion	Cystic lesion
Type I	Type CE1	Unilocular anechoic cystic lesion with double line sign	Active stage
Type III	Type CE2	Multiseptated rosette like/honeycomb cyst	Active stage
Type II	Type CE3	Cyst with detached membranes (water lily sign) Cyst with daughter cyst in solid matrix	Transitional stage
Type IV CE4	Type CE4	Cyst with heterogenous content; no daughter cysts	Inactive stage
Type V	Type CE5	Solid and calcified wall	Inactive stage

The approach to the treatment of hydatid disease is complex and depends on cystic characteristics and stage, available surgical and medical expertise and equipment, and patients’ compliance with long-term monitoring [15]. The cyst type, size, location, and the presence or absence of complications guide treatment options [15]. Radical cyst resection, percutaneous cyst aspiration and reaspiration, and medical treatment with mebendazole or albendazole constitute the mainstay of treatment [8,15].

Literature review

Twenty-three case reports were retrieved searching the PubMed and Google Scholar databases that described the occurrence of primary isolated mesenteric hydatid cyst (Table 3). The patients were of variable ages with a wide age range extending from three to 52 years. The exposure history was only mentioned in five papers, in which only one reported a negative exposure history [14] similar to the patient in our case report. Whereas exposure to farm animals, cattle, and sheep was reported in three articles [3,5,11], exposure to dogs was noted in one study [16].

**Table 3:** Review of reported cases of primary mesenteric hydatid cysts Y, years; US, ultrasound; WHO, World Health Organization; CT, computed tomography scan; M, male; m, month; URK, unremarkable; F, female; RLQ, right lower quadrant; IgG, immunoglobulin G; BID, twice daily; d, days; LUQ, left upper quadrant; PAIR, puncture (P), aspiration (A), instillation (I), reaspiration (R); SB, small bowel; h, hours; IHA, indirect hemagglutination assay; ELISA, Enzyme-Linked Immunosorbent Assay; HC, hydatid cyst.

Author	Sex	Age (y)	Symptoms	Duration of Symptoms	Lab findings	Hydatid Lab tests	US findings	WHO US stage	Gharbi’s classification	CT findings	Local mass effect	Rupture	Surgical procedure	Protoscolicidal agent	Location within the mesentery	Medical treatment	Method of diagnosis	Follow-up
Tawashi et al, 2023 [1]	M	9	Umbilical abdominal pain, severe mucoid diarrhea, nausea, vomiting, and fever	0 d	Leucocytosis; neutrophilia; anemia	-	1^st ^US: thickened appendicial wall with mild free fluid in the abdomen and pelvis 2^nd ^US: dilated bowel loops with a cystic formation (7cm) in the umbilical area	-	-	-	No	Yes	Resection of germinal wall and daughter cysts + partial wall excision	Hypertonic saline 30%	Mesoileum	Albendazole for 6 months	Intraoperative	-
Sheikh et al. 2019 [2]	F	46	Progressively growing abdominal lump	6 m	Leucocytosis; eosinophilia (16%)	-	Hypoechoic lump (20x18x15) cm with internal echoes	CE1	Type I	Cystic mass (21x20x11) cm with calcifications, extending from epigastric region to the gallbladder fundus and inferiorly indenting the sigmoid colon	Sigmoid colon indentation	No	Surgical excision	No	-	-	Histopathological	-
Kulkarni, 2013 [3]	M	60	Abdominal pain, which worsened after heavy meals and sleep, nausea following meals by 2-3 h, and fever	15 d	Leucocytosis; neutrophilia; anemia; eosinophilia (8%)	Serology IHA: + ELISA: +	Multiple hypoechoic cysts in the small intestine	CE	Type I	-	No	No	Cystectomy + Partial jejunal resection	Cetrimide	Mesojejenum	Albendazole 400 mg BID for 3 months	Intraoperative	No recurrence after 12 months
Kushwaha et al. 2012 [4]	M	24	Exertional suprapubic pain	1 y	Eosinophilia (12%); anemia	ELISA (+)	Large triple-layer-walled cystic lesion with cysts within cyst, and multiple small cysts attached to wall	CE2	Type III	-	No	No	PAIR + Cystectomy	Povidone iodine 10%	Mesojejenum	Albendazole 400 mg BID for 1 month	Radiological	Well after follow-up
Agarwal et al. 2013 [5]	F	30	Intermittent vague right-sided abdominal pain and discomfort; low-grade fever	1 y	Eosinophilia (8%); anemia	-	Multi-loculated Hypo-echoic mass (10x12) cm adhered to the right adnexal region, caecum, and appendix	CE2	Type III	-	No	No	Cystectomy + appendectomy	No	Mesoappendix	Albendazole 400 mg BID for 1 month	Histopathological	No recurrence after 6 months
Ranjan et al. 2023 [6]	F	39	Dull aching RLQ pain	2 m	Eosinophilia (5%)	Echinococcus IgG (+)	Ill-defined cystic lesion (7.8x4.9) cm with multiple internal septations	CE2	Type III	Well-defined cystic lesion (9.2x8.6) cm with multiple internal septations	No	No	Cystectomy	No	Mesenteric	Albendazole 400 mg Bid for one month	Intraoperative	No recurrence after three months
Adelyar et al, 2021 [7]	F	40	Progressive LUQ and left flank pain	3 y	Eosinophilia (5%) Anemia	No	Well-defined large cystic lesion in the LUQ	CL	-	Well-defined thick-walled cystic lesion at LUQ	Moderate hydronephrosis and hydroureter	No	PAIR + cystectomy	Hypertonic saline 20%	Mesojejunum	Albendazole 400 mg bid for 1 month	Intraoperative	Asymptomatic after 3 years
Sebahiga et al. 2024 [8]	M	14	Abdominal mass	6 m	URK	-	Well-limited avascularized anechoic formation, without septum or endocystic vegetations	CL	-	Non-enhancing periumbilical cystic formation (4x5x5) cm in contact with bowel loops	No	No	Cystectomy + Partial ileal resection and primary anastomosis	No	Mesoileum	No	Histopathological	-
Reddy 2021 [9]	M	62	Intermittent abdominal pain and distention; recurrent vomiting; nausea	3 m	URK	Serology (+)	Well-defined cystic intraperitoneal mass (7x5) cm with multiple internal septations and no evidence of solid component	CE2	Type III	-	No	No	PAIR + Subtotal cystectomy	Hydrogen peroxide	SB mesentery	No	Radiological	No recurrence after two years
De 2009 [10]	F	56	Acute RLQ pain with nausea, vomiting, and fever	Acute	Anemia; leucocytosis; neutrophiia; eosinophilia (19%)	-	Multiseptated cyst (5.2x2.5 cm) with a honeycombed appearance in the right iliac fossa	CE2	Type III	-	No	No	Cyst Enucleation	No	Mesoappendix	Albendazole 400 mg BID	Radiological	No recurrence
Bagul and Bagul 2009 [11]	M	3	Intermittent abdominal pain; weight loss of 6 kg over 3 months	15 d (pain) 3 m (weight loss)	URK	-	Multiple cystic areas in the central abdomen, with cysts measuring 2 mm to 8 cm with multiple loculi	CE2	Type III	-	No	No	Total resection of multiple HC + Partial ileal resection	No	Mesojejenum and mesoileum	Albendazole 10mg/kg/day for 8 months	Radiological	No recurrence after 5 years (by US)
Mittal et al. 2012 [14]	F	35	Dull pain in the lower abdomen	2 y	URK	-	Large multiseptated mass (11x7) cm in the right adnexa	CE2	Type III	-	Deviation of the uterus	No	Cystectomy + Total abdominal hysterectomy	No	-	Albendazole 10mg/kg/day for 6 months	Histopathological	-
BouK’Hil et al. 2019 [16]	M	7	Abdominal pain and right flank distension	2 m	-	Serology (+) (1/160)	Well-defined oval-shaped mass in the right flank with partially calcified wall, hyperechogenic and heterogeneous with fluid material and detached membranes	CE 3a	Type II	Round non-enhancing cystic formation (3.5x4.2x3.8) cm	No	No	Surgical excision	No	-	No	Radiological	No recurrence (by US) after 12 months
Gün et al. 2007 [17]	M	12	Abdominal pain and rash after blunt abdominal trauma	-	Anemia; leucocytosis	-	Cystic mass of the abdominal mesentery	CL	-	Lesion in the intestinal mesentery (3x4x4) cm	No	Yes	Total cystectomy	No	Mesoileum	Albendazole	Histopathological	No recurrence
Kusaslan et al. 2007 [18]	M	19	Diffuse abdominal pain, nausea, vomiting, and fever	3 h	Leucocytosis	-	Cystic mass (8x10) cm in the lower quadrant with a mild amount of fluid in the abdominal cavity	CL	-	-	No	Yes	Partial cystectomy	Povidone iodine 10%	Mesoileum	Albendazole 10mg/kg for 6 months	Intraoperative	-
Jatal et al. 2023 [19]	M	30	Abdominal pain and a palpable lump in the right iliac fossa	-	URK	Casoni test (-)	Well-defined anechoic, double-walled cystic mass (10x8) cm	CE1	Type I	-	No	No	Subtotal cystectomy	Povidone iodine 10%	-	Albendazole for 6 months	Intraoperative	No recurrence after 1 year
Najih et al. 2012 [20]	M	43	Intermittent abdominal pain, abdominal distension, and recurrent nausea and vomiting	10 m	URK	Serology (-)	Heterogeneous intraperitoneal mass (9x5) cm	CE4	Type IV	Intraperitoneal cyst (12x7) cm with wall calcifications and connection to the duodenum	No	No	PAIR + Subtotal cystectomy	Hydrogen peroxide	Mesoileum	No	Intraoperative	No recurrence after 34 months
Duzkoylu and Kucuk 2021 [21]	M	62	Abdominal distension; intermittent abdominal pain	5 y	-	-	Cystic lesion (18x15) between the supra-umbilical region and pelvis	CL	-	Cystic lesion (19x15) cm originating from 3^rd^ part of duodenum	No	No	Cystectomy	No	Proximal SB mesentery	Albendazole 800 mg/d for 3 months	Histopathological	-
Singh 2018 [22]	M	13	Gradually enlarging umbilical abdominal mass	6 m	-	-	-	-	-	Well-circumscribed cystic lesion (6.6x4.4x5) cm displacing bowel loops posterolaterally. No internal septations or daughter cysts	Bowel loops displacement	No	Total cystectomy	No	-	-	Histopathological	-
Kerkeni et al. 2015 [23]	M	5	Abdominal mass	-	-	-	Well-defined anechoic cystic mass with detached floating membrane	CE3a	Type II	-	Inguinal ring obstruction	No	PAIR + Partial cystectomy	Hypertonic saline 20%	Sigmoid mesocolon	Albendazole 10mg/kg/day for 3 months	Radiological	No recurrence after 8 months
Yagmur et al. 2015 [24]	F	19	Abdominal pain and nausea	1 y	URK; no eosinophilia	-	Cyst located between the right lobe of the liver and the right kidney (8.5x5.3) cm	-	-	Cyst bordering the right kidney (8x5) cm	No	No	PAIR + Pericystectomy	Povidone iodine	-	Albendazole 10mg/kg/day	Radiological	-
Ahmad et al. 2010 [25]	M	25	Diffuse abdominal pain, most on RLQ	-	Anemia; no eosinophilia	-	-	-	-	-	Narrowing of the terminal ilium with fibrosis, contracture, and cecal distortion (pulled up)	No	No details	No	Ileocecal mesentery	-	Histopathological	-
Astarcioglu et al. 2001 [26]	M	61	Abdominal pain, distension, obstipation, vomiting, and fever	3 d	Leukocytosis	ELISA (+) 1/256	Dilated colonic segments and moderate amount of fluid in the abdominal cavity	-	-	-	Left colon obstruction	No	Total resection of multiple HC + Hartmann’s procedure	No	Mesosigmoid	Albendazole 200 mg/day	Intraoperative	No recurrence after 4 months

The literature reveals a wide range of presentations and clinical pictures depending on the location of the hydatid cyst and the local mass effect it produced. Abdominal pain, comparable to the chief complaint of our patient, constituted the majority of the reported complaints. A few presented with abdominal mass or lump. The location of the abdominal pain was variable and mostly dependent on the location of the hydatid cyst (right upper and lower quadrants, umbilical, right and left flanks, diffuse abdominal, and suprapubic). Many patients reported nausea and vomiting. Fever was reported in seven cases. An urticarial rash was described by Gün et al. in a 12-year-old patient following blunt abdominal trauma, which was related to a rupture of a not-yet-diagnosed mesenteric hydatid cyst [17].

Similar to the case presented in this report, the literature reported symptoms that were for the most part intermittent and progressive, with many patients reporting nonspecific symptoms for prolonged periods prior to the definitive diagnosis. The duration was varied and, excluding acute presentations, ranged from a minimum of fifteen days to a maximum of five years. Likewise, our patient has been experiencing nonspecific abdominal complaints for the past four years. Acute presentation was reported in four papers, three of which were ruptured mesenteric cysts [1,10,17]. The article by Kusaslan et al. reported an onset of acute abdominal pain and fever without trauma or triggering event that was later discovered to be a ruptured mesenteric cyst [18]. Abdominal blunt trauma was the inciting event in the article reported by Gün et al. [17]. The case by Tawashi et al. documented a ruptured mesenteric hydatid cyst and acute abdomen triggered by a preceding appendectomy procedure [1]. And finally, De reported an acute presentation of a ruptured mesenteric hydatid cyst without a clear triggering event [10]. The presence or absence of eosinophilia was reported in nine articles, seven of which documented an elevation. Leukocytosis and anemia were among the other reported laboratory changes. Since the occurrence of mesenteric hydatid disease is rather rare, and many cases were diagnosed intraoperatively or histopathological, only eight reports used echinococcosis-specific diagnostic tools. All the utilized tests were serological, except for Jatal et al. who used the Casoni skin test with a negative result [19]. Only Najih et al. disclosed a negative serology result [20].

The imaging modalities performed were either ultrasound or CT scan or both. The cyst was detectable on both modalities and in various stages of development, except in the case by Tawashi et al. where it was missed on ultrasound [1]. Many clinical presentations were dependent on the mass effect the cyst produced. Hydronephrosis and hydroureter, small and large bowel compression or obstruction, uterine deviation, and inguinal ring obstruction were among the reported mass effects. All the reported cases were treated surgically and combined surgical and medical treatment with albendazole was provided in 16 articles. Within the mesentery, most of the cysts were located in the small bowel mesentery, specifically the mesoileum. Other reported sites were the mesoappendix and the ileocecum. A good no-recurrence prognosis was reported in all the papers that provided a follow-up.

## Conclusions

The occurrence of hydatid disease in the mesenteric region as a primary event is a rare entity and results from the direct hematogenous or lymphatic dissemination of the oncospheres bypassing the liver and lungs. The most common site within the mesentery is the small bowel mesentery, but virtually any mesenteric region can become affected. Mesenteric hydatid cysts can grow to large sizes before official diagnosis, producing progressive and prolonged symptoms. The duration, size, and mass effects are key factors in the clinical presentation. It is important to differentiate mesenteric hydatid cysts from other cystic-like lesions of the abdomen. Surgery constitutes the definitive treatment.

## References

[1] Tawashi K, Tawashi Y, Naqoula S (2023). A hydatid cyst in mesentery complicated with appendicitis in nine-year-old child. J Pediatr Surg Case Rep.

[2] Sheikh T, Sahu S, Karale S (2019). Hydatid cyst in mesentery: rare and unusual site of occurrence. Int J Health Sci Res.

[3] Kulkarni N (2013). Intestinal hydatidosis in unusual location: a case report. Int J Case Rep Images.

[4] Kushwaha JK, Gupta R, Mohanti S, Kumar S (2012). Primary mesenteric hydatid cyst. BMJ Case Rep.

[5] Agarwal D, Huda F, N A, Awasthi S (2013). A hydatid cyst of the appendix which mimicked a tubo-ovarian mass: a case report and review of the literature. J Clin Diagn Res.

[6] Ranjan R, Kumari K, Milan KC, Awale L, Khaniya B (2023). Mesenteric hydatid cyst mimicking an ovarian cyst - a case report. Int J Surg Case Rep.

[7] Adelyar MA, Hesham S, Walizada K, Mushkani EA, Saadaat R (2022). Primary mesenteric hydatid cyst; a rare manifestation of hydatid disease through a case report and literature review. Int J Surg Case Rep.

[8] Sebahiga C, Touré S, Aballa N, El Ouafi K, Fouraiji K, Oulad Saiad M (2024). Primary mesenteric hydatid cyst in pediatric patients: case report and literature review. SAS J Surg.

[9] Reddy R Concurrent occurrence of primary mesenteric hydatid cyst with multiple calcified splenic granulomas: a rare presentation. Apollo Medicine.

[10] De U (2009). Rare primary extrahepatic intra-abdominal hydatid cysts. Trop Doct.

[11] Bagul A, Bagul M (2009). Primary ileal mesenteric hydatidosis: a rare cause of colicky abdominal pain in childhood. J Paediatr Child Health.

[12] Maleki F, Akhlaghi L, Tabatabaie F (2023). Evaluation of hydatid cyst antigen for serological diagnosis. Med J Islam Repub Iran.

[13] Samsami M, Qaderi S, Zebarjadi Bagherpour J, Lucero-Prisno DE 3rd (2021). A case report of primary isolated extrahepatic hydatid cyst of the soft tissues of the breast and thigh. Int J Surg Case Rep.

[14] Mittal S, Taneja BK, Goel A, Puri M, Pandey P (2012). Mesenteric hydatid cyst: an unusual presentation. Korean J Obstet Gynecol.

[15] Brunetti E, Kern P, Vuitton DA (2010). Expert consensus for the diagnosis and treatment of cystic and alveolar echinococcosis in humans. Acta Trop.

[16] BouK’Hil KS, BeKKat-BerKani D, MeSSaDi W (2019). Kyste hydatique mésentérique primitif (in French). El Hakim J.

[17] Gün F, Devecioglu D, Salman T (2007). Traumatic rupture of hydatid cyst with unusual localization without liver involvement: a case report. Eur J Pediatr Surg.

[18] Kusaslan R, Sahin DA, Belli AK, Dilek ON (2007). Rupture of a mesenteric hydatid cyst: a rare cause of acute abdomen. Can J Surg.

[19] Jatal SN, Jatal S, Ingle S (2023). Primary mesenteric hydatid cyst - a rare case report. Asian J Case Rep Surg.

[20] Najih M, Chabni A, Attoulou G, Yamoul R, Yakka M, Ehirchiou A, AlKandry S (2012). Isolated primary hydatid cyst of small intestinal mesentery: an exceptional location of hydatid disease. Pan Afr Med J.

[21] Duzkoylu Y, Kucuk AI (2021). A benign rare intraabdominal lesion: primary giant mesenteric hydatid cyst. J Clin Images Med Case Rep.

[22] Singh BK (2018). Mesenteric hydatid cyst. Nepalese J Radiol.

[23] Kerkeni Y, Sahli S, Gasmi M, Sghairoun N, Hamzaoui M (2017). A rare cause of recurrent vaginal hydrocele: herniating mesenteric hydatid cyst. Iran J Parasitol.

[24] Yagmur Y, Babur M, Gumus S, Can MA (2015). Laparoscopic treatment of primary mesenteric hydatid cyst. J Gastroenterol Hepatol Res.

[25] Ahmad SS, Hassan MJ, Anees A, Rahman K, Zaheer S, Qaseem SM (2010). Hydatid disease of the intestine manifesting as acute abdomen: a case report. Am J Gastroenterol.

[26] Astarcioglu H, Koçdor MA, Topalak O, Terzi C, Sökmen S, Ozer E (2001). Isolated mesosigmoidal hydatid cyst as an unusual cause of colonic obstruction: report of a case. Surg Today.

